# Improving patient information for telemonitoring in chronic heart failure

**Published:** 2012-06-15

**Authors:** Arina Burghouts

**Affiliations:** NICTIZ, Amsterdam, The Netherlands

**Keywords:** telemonitoring, chronic heart failure, interoperability, profiles

## Abstract

**Background:**

More and more people suffer from heart failure and the expectation is that this number will only increase the coming years. Innovations are needed to keep healthcare accessible as well as affordable. Telemonitoring is one of the promising innovations that can be deployed for making the care for heart failure patients safer and more efficient. Nevertheless, the use of these eHealth solutions are not yet in proportion to our objective. There are many reasons for this situation in terms of funding, acceptance, questions about liability, etc. Another very important reason is the lack of interoperability: there is no interaction or information exchange between different systems. This leads to a situation in which information is not, or not in time, available to the care provider. A heart failure patient using telemonitoring measures his body weight, his blood pressure and answers some questions on a daily basis. Based on these data, the care providers in the hospital are able to monitor the health status of the patient over a distance. However, care providers lack access to all information on the patient in one application. The telemonitoring information can be found in the telemonitoring system, whereas the other medical information (medication overview, medical history, etc.) is stored in the hospital information system or electronic patient record. As a result, not all patient information is available in one system or it has to be copied manually, with all the consequences that can entail.

**Aims and objectives:**

The aim of this project is to improve the information exchange and to stimulate the use and acceptance of telemonitoring. Nictiz initiated assembling all stakeholders to develop interoperability profiles that will improve the information exchange.

**Methods and results:**

To enable interoperability, standards are a required but not sufficient condition. It is also necessary to agree on how those standards are applied to support specific care processes and to exchange the correct information at the correct moment. This can be achieved by developing interoperability profiles. In these profiles agreements between all stakeholders are recorded on process, information, application, and technical level. Starting point was the problem on information exchange described above and the needs and the interests of the stakeholders. Based on this specific use case, health care professionals, patient representatives, IT suppliers, and insurers collaborate to make agreements about interoperability between the telemonitoring system and the electronic patient record used in the hospital. This results in functional and technical design specifications, based on the Continua Design Guidelines. These profiles will be implemented in the relevant applications, resulting in an information exchange between the telemonitoring systems and the electronic patient record systems in a standardized way.

**Conclusion:**

With the use of interoperability profiles defined by all stakeholders, the telemonitoring data are available in the electronic patient record of the heart failure patient used in the hospital. In this way, all information is easily available for the care providers, thereby making the care for heart failure patients safer and more efficient.

